# The ARL2 GTPase Is Required for Mitochondrial Morphology, Motility, and Maintenance of ATP Levels

**DOI:** 10.1371/journal.pone.0099270

**Published:** 2014-06-09

**Authors:** Laura E. Newman, Cheng-jing Zhou, Samatha Mudigonda, Alexa L. Mattheyses, Eleonora Paradies, Carlo Marya Thomas Marobbio, Richard A. Kahn

**Affiliations:** 1 Department of Biochemistry, Emory University School of Medicine, Atlanta, Georgia, United States of America; 2 Department of Pediatrics, Emory University School of Medicine, Atlanta, Georgia, United States of America; 3 Department of Cell Biology, Emory University School of Medicine, Atlanta, Georgia, United States of America; 4 Consiglio Nazionale delle Ricerche Institute of Biomembranes and Bioenergetics, Bari, Italy; 5 Department of Biosciences, Biotechnology and Biopharmaceutics, University of Bari, Bari, Italy; National Institute of Environmental Health Sciences, United States of America

## Abstract

ARF-like 2 (ARL2) is a member of the ARF family and RAS superfamily of regulatory GTPases, predicted to be present in the last eukaryotic common ancestor, and essential in a number of model genetic systems. Though best studied as a regulator of tubulin folding, we previously demonstrated that ARL2 partially localizes to mitochondria. Here, we show that ARL2 is essential to a number of mitochondrial functions, including mitochondrial morphology, motility, and maintenance of ATP levels. We compare phenotypes resulting from ARL2 depletion and expression of dominant negative mutants and use these to demonstrate that the mitochondrial roles of ARL2 are distinct from its roles in tubulin folding. Testing of current models for ARL2 actions at mitochondria failed to support them. Rather, we found that knockdown of the ARL2 GTPase activating protein (GAP) ELMOD2 phenocopies two of three phenotypes of ARL2 siRNA, making it a likely effector for these actions. These results add new layers of complexity to ARL2 signaling, highlighting the need to deconvolve these different cell functions. We hypothesize that ARL2 plays essential roles inside mitochondria along with other cellular functions, at least in part to provide coupling of regulation between these essential cell processes.

## Introduction

GTPases in the RAS superfamily have emerged not only as regulators of many specific signaling and metabolic pathways but also provide integration between pathways through the use of common GTPases or effectors. ADP-ribosylation factor-like 2 (ARL2), within the ARF family of ∼30 genes/proteins in mammals, is one such regulator and is the focus of this study. ARL2 is highly conserved in eukaryotes and ubiquitously expressed [1]. It plays roles in both the regulation of tubulin folding and microtubule destruction [2], [3], and is found in cytosol tightly bound to the tubulin specific co-chaperone, cofactor D, which shares those activities. Mutations in both ARL2 and cofactor D have been identified in a number of genetic screens linked to microtubules in model organisms that include *S. cerevisiae*, *S. pombe*, *A. thaliana*, and *C. elegans* [4], [5], [6], [7], [8], [9]. Using stable variants of human breast adenocarcinoma lines, Beghin, et al [10],[11] showed that ARL2 protein content influences cell morphology (or cell spreading), soluble tubulin pools, microtubule dynamics, cell cycle progression, and chemosensitivity. ARL2 has also been proposed to regulate aspects of STAT3 signaling through actions in the nucleus [12]. In addition to its localizations in the nucleus and cytosol, we have shown that ARL2 is also localized inside mitochondria, as ARL2-specific antibodies localized it to mitochondria in a number of different cell lines and tissues [13]. Thus, we set out to examine its role(s) in mitochondria both to better understand its cellular functions and also to provide potential insights into how these different essential cell roles may be integrated, as defects in any of the processes linked to ARL2 dysfunction are hallmarks of the transformed state, cancer, and a host of human diseases.

The mitochondrial pool of ARL2 is estimated to be ∼10% of total cellular ARL2, though that percentage also appears to be subject to regulatory influences [13], [14]. We earlier concluded that ARL2 was present in the inner membrane space (IMS) of mitochondria and a recent proteomic study confirmed our conclusions, using spatially restricted enzymatic tagging in human embryonic kidney cells [15]. Our previous studies also localized the ARL2 effector Binder of ARL2 (BART, or ARL2BP) [13], [16] to mitochondria. Binding of ARL2 to BART is promoted by activation of the ARL2, via its binding to GTP. The ARL2(GTP)-BART complex binds the adenine nucleotide transporter, ANT1, in an *in vitro* gel overlay assay [13] though the consequences of this association to ANT activity are unknown. Thus, while ARL2 clearly localizes to mitochondria, its function(s) there are poorly understood. The ARF and RAS families of GTPases are predicted to have arisen in prokaryotes [17] and thus specific roles in mitochondrial biology may be among the most ancient signaling pathways known to have survived the emergence of eukaryotes. Therefore, a role for a nuclear encoded regulatory GTPase inside mitochondria is expected to provide potentially important insights into both mitochondrial and evolutionary biology.

The presence of ARL2 in multiple cellular locations and its proposed regulation of multiple cellular processes are consistent with other RAS superfamily and ARF family members displaying such characteristics. Indeed, the challenge to researchers has changed from earlier attempts to identify the signaling pathway regulated by a GTPase to deconvolution of the multiple processes that lie downstream. In efforts to develop models for ARL2 signaling pathways, we purified the only known ARL2 GAPs, ELMOD1-3 [18]. ELMOD proteins are highly conserved in eukaryotic evolution, predicted to be present in the last eukaryotic common ancestor and the defining ELMO domain was shown to be the ARL2 GAP domain [19]. Roles for at least two of the three ELMOD proteins in deafness in mammals [20], [21] further highlight the need to understand ARL2 regulation and cellular functions. Similarly, it is common in GTPase families for each protein to have close paralogs that may share overlapping functions. Therefore, it is important to also discriminate between roles for each GTPase within a family as new functions emerge. The closest ARL2 paralog is ARL3, which shares 53% identity with ARL2, which has distinct functions [22], [23], [24], [25], [26] yet is also a substrate for ELMODs as GAPs [18].

As is common in GTPase research, we took advantage of previously described [22] dominant activating (ARL2[Q70L] and inactivating (ARL2[T30N]) point mutants to test for effects of excessive or loss of ARL2 activities. These mutants are homologous to dominant activating (Q61L) or dominant inactivating (S17N) mutants of Ras proteins [27], [28], [29] or Q71L and T31N mutants of ARFs (e.g., see Zhang, et al [30]). Dominant activating mutants are persistently active because the mutated glutamine is involved in hydrolysis of the γ-phosphate of bound GTP and its loss leads to reduced ability to hydrolyze GTP in the presence of the GTPase activating protein (GAP). The dominant inactivating (or dominant negative) mutants are thought to bind their cognate guanine nucleotide exchange factor (GEF) but bind GTP poorly, leading to a more stable GTPase-GEF complex that prevents activation of endogenous GEF substrate(s). We also employ short interfering RNAs (siRNAs) to knock down protein expression in cultured cells to identify phenotypes resulting from the deficiency of the specific protein under study. Because knockdown of a protein can be incomplete and varies with time after expression or transfection of the siRNA, it is not uncommon to see different phenotypes, or more severe phenotypes, with expression of the dominant negative mutants. Therefore, comparing effects of each approach has also proven instructive due to their distinct mechanisms of action. Here we document roles for ARL2 in mitochondrial functions by comparing phenotypes of mitochondria in cells either deficient for ARL2 or expressing dominant negative ARL2 and develop novel models for ARL2 actions at mitochondria. Particular attention was paid to resolve effects of ARL2 on microtubules from those on mitochondria to allow current and future efforts to determine mechanisms of each as well as develop models for integration of these different essential functions.

## Materials and Methods

### Cell Culture

Human cervical carcinoma (HeLa) were grown in DMEM supplemented with 10% fetal bovine serum (Invitrogen, Carlsbad, CA) and 2 mM glutamine at 37°C in the presence of 5% CO_2_. Cells were obtained from ATCC.

### Antibodies

Affinity-purified rabbit, polyclonal ARL2 (R-86336) or BART (R-46712) antibodies were prepared as described in Sharer *et al.* [13]. Monoclonal antibodies directed against cytochrome-c and TOM20 were purchased from BD Transduction Laboratories (Lexington, KY). Mouse monoclonal antibodies to actin and α tubulin were purchased from Sigma (St. Louis, MS). A rabbit polyclonal antibody to GFP was purchased from Abcam.(Cambridge, MA). Mouse monoclonal ADI-SPA-807, directed against HSP60, was purchased from Stressgen (now Enzo Life Sciences). To probe electron transport chain complexes, we used a cocktail of 5 antibodies that recognized NDUFA9 (Complex I), core protein 2 (Complex II), UQCRC2 (Complex III), subunit IV (Complex IV), and subunit alpha (Complex V), purchased from Mitosciences (catalog # MS603). ARL3 [22] and ELMOD2 antibodies were raised in rabbits against purified, bacterially expressed human proteins and characterized in our lab. All such antibodies (including those directed against ARL2) at a minimum react with the purified antigen as well as a band of the correct electrophoretic mobility in lysates from cells over-expressing the antigen in immunoblots, while the pre-immune sera do not. Also, the appropriate band is effectively competed by prior incubation of the antiserum with antigen.

### Plasmids

Plasmids directing expression of human ARL2, ARL2[T30N], or ARL2[Q70L] in the pcDNA3.1 backbone have been described previously [22]. Site directed mutagenesis was used to generate the K71R mutation in the same vector; work was performed at the Emory Cloning Core. Each was sequence verified. GFP-DRP1[K38A] [31] was a gift from Dr. Edward Bampton (University of Leicester, UK).GFP-Mito and DsRed-Mito were provided by Dr. James Zheng (Emory University). The source of RAB5-GFP and RAB7-GFP plasmids were Dr. Allan Levey (Emory University) and Dr. Richard Pagano (Mayo Clinic, MN), respectively. The coding sequence of bovine ANT1/AAC1 (SLC25A4) was amplified by PCR from total bovine heart cDNA. The oligonucleotide primers were synthesized corresponding to the extremities of the coding sequence (accession NM174658.2), with *Nde*I and *Hind*III sites added. The amplified product was cloned into pET21b for expression in *E. coli* and the sequence was verified.

### siRNAs

siRNAs were obtained from Dharmacon (Lafayette, CO), with the exception of individual ELMOD2 siRNAs, which were obtained from Sigma (St. Louis, MS). Catalog numbers are as follows: ARL2 siRNA #1 J-011585-05 (CAACCAUCCUGAAGAAGUU), ARL2 siRNA #2 J-011585-06 (GAGCAACCCUCCUCAUCUU), BART siRNA #1 J-013074-04, #2 J-013074-12, Arl3 SmartPool L-011813-00, ELMOD2 SmartPool L-016104-00. Individual ELMODs siRNAs were ordered from Sigma with the following modifications: 5′ amine C6, 3′ PHOS, 3′ dT overhang, and 2′ verite. The sequences were (#2) AUAUAAAUUCUGUUGAAUG and (#3) UCAAUUUAAUACAAAUACC, directed against the 3′ UTR.

### Transfection with siRNA and plasmids

Cells were grown in six-well culture dishes to 70–90% confluence. Plasmids (2 µg of pcDNA3.1 or pcDNA3.1 carrying indicated inserts and Mito-GFP; 2∶1 ratio) were co-transfected using LipofectAMINE and Plus reagents (Invitrogen) according to the manufacturer's instructions. siRNAs (25 nM) were transfected into HeLa cells using Dharmafect transfection reagent #1 (Dharmacon, Lafayette, CO) following the company's instructions. For some experiments, transfections were performed sequentially first with plasmid DNA followed 5 hours later by transfection of the ARL2 siRNA, to minimize cell toxicity evident from co-transfections. We consistently observed at least 70% transfection efficiency, using expression of fluorescence-tagged proteins to score.

### Immunofluorescence analyses

Cells were grown on Matrigel-coated coverslips. For imaging mitochondria and ARL2, cells were fixed in 4% paraformaldehyde followed by permeabilization with 0.1% (v/v) Triton X-100 in PBS. Incubation with primary antibodies was carried out in PBS containing 1% (w/v) BSA, at 4°C overnight. Secondary antibodies (Alexa fluorophores, Invitrogen) were incubated in the same buffer for 1 hour at room temperature. Cells were examined using a Zeiss LSM 510 microscope (Thornwood, NY) using 100× objective with laser excitation at 488 or 543 nm. To visualize microtubules, we used PHEMO fixation (3.7% (v/v) paraformaldehyde, 0.05% (v/v) glutaraldehyde, 0.5% (v/v) Triton X-100 in 68 mM PIPES, 25 mM HEPES, 15 mM EGTA, 3 mM MgCl_2_, 10% (v/v) dimethylsulfoxide, pH 6.8) for 10 minutes at room temperature, as described in Mabjeesh *et al* [32]. Cells were then stained and imaged as described above.

The sub-mitochondrial location of ARL2 was investigated by the use of differential permeabilization of fixed HeLa cells with increasing concentrations of digitonin, as described previously [33], [34]. Briefly, cells were used at ∼70% confluence. Prior to fixation, cells were treated with 0.1% (w/v) saponin in growth media for 1 min. on ice. This pre-permeabilization fixation decreased background, cytosolic staining of ARL2, BART, and ELMOD2, making endogenous mitochondria-associated protein easier to detect. Cells were then immediately fixed in 4% paraformaldehyde for 15 minutes at room temperature. Fixed cells were then permeabilized with 0.2 or 1.0 mg/mL digitonin in PBS for 10 minutes at room temperature, and immunofluorescence was performed as previously described.

### Live cell imaging

Cells were imaged for 8 hours, starting 24 hours after transfection, for both siRNA and overexpression experiments. Cells already displaying mitochondrial fragmentation at the beginning of the imaging period were excluded from analysis. Live cell imaging was performed using a Nikon A1R confocal microscope, enclosed in a heating chamber at 37°C with 5% CO_2_ perfused over the plate holder. Cells were plated on 35 mm live-cell imaging dishes and imaged using a 63X (NA 1.4) objective. Time-lapse images were taken every 20 seconds over a period of 8 hours or as indicated. For experiments using only GFP-labeled proteins, the fluorophore was excited using the 488-nm line of an Ar ion laser. For time-lapse sequences where dual labels were used (Lysotracker red; Molecular Probes, OR; and GFP-Mito), the fluorophores were sequentially excited with the 488 and 543 laser lines and emission signals were separated using an electronically controlled emission discrimination filter with the appropriate filter sets. To monitor lysosome movements, LysoTracker Red (Molecular Probes) was added to cells 30 minutes prior to imaging. To visualize early or late endosome motility we expressed GFP-RAB5 or GFP-RAB7, respectively.

### Mitochondrial Morphology Assays

Alterations in mitochondrial morphology were generally observed as changes in the length of the organelle and the distribution of mitochondria in the cell. Acquired images of cells stained with mitochondria markers (cytochrome c, TOM20, HSP60, etc.) were analyzed using NIH Image J software (Wayne Rasband, NIH). Multinucleated cells were excluded from this analysis. For quantitative analyses, cells were classified according to their mitochondrial morphology into four different groups: tubular (if most mitochondria were filamentous); perinuclear (mitochondrial aggregates were located near the nucleus); fragmentation (all mitochondria were fragmented and no filamentous mitochondria were found) and intermediate (a mixture of short tubular and fragmented forms). For each experiment at least 200 cells were scored from three different experiments in the analysis and values are represented as means ± one standard deviation (1 SD) of three independent experiments. Statistical analysis was determined using Student's *t* test. A value of p<0.05 was considered significant unless otherwise indicated.

For 3D imaging, 20–22 Z-sections of mito-GFP-expressing cells were obtained at 0.2-µm intervals through the entire cell using a piezo-electric focus motor mounted onto the objective lens of a Nikon Widefield microscope. Subsequent image restoration was achieved with Huygens Essential (SVI, Netherlands) deconvolution software, using the deconvolution algorithm with a computed point spread function. Image analysis and processing was performed with ImageJ (NIH; http://rsb.info.nih.gov/ij).

### Tracking of Mitochondrial Movements

Images were subjected to motility analysis by generating kymographs using the plug-in MTrackJ (Meijering, E., University Medical Center of Rotterdam, Netherlands; http://www.imagescience.org/meijering/software/mtrackj/) and ImageJ (NIH) (Abramoff et al., 2004). Kymographs of mitochondria in time lapse images from HeLa cells expressing mito-GFP were produced using ImageJ, as described previously [35]. Motile mitochondria were identified as moving objects in movies and diagonal lines in the kymographs. The velocity of the motile mitochondria was extracted from the kymographs. The displacement of a mitochondrion from one frame to the next was converted from pixels to real distances by calibrating the *x-y* axes of the analyzed images in MTtrackJ. For tubular mitochondria, the ends were tracked whereas for fragmented mitochondria the center was tracked. The obtained x-y-t tracking coordinates of mitochondrial movements were analyzed with Excel-based software. The start of a run was defined by a minimal displacement of 0.1 µm/sec. Mitochondria were classified as either moving or stationary based on whether they achieved a displacement of >1 µm and also by the vertical straight lines in the kymographs. Tracks of the ends of different mitochondria in a one hour period are shown in different colors and explained in the legends. All the values are means ±1 standard deviation of randomly selected mitochondria; at least 8 individual cells were analyzed.

### Determination of cellular and mitochondrial ATP

ATP concentrations were measured using the ENLITEN ATP Assay Bioluminescence Detection Kit (Promega), according to the manufacturer's instructions. Briefly, HeLa cells were grown in six well plates (3 wells/condition) and transfected as described above. Cells were seeded to ensure densities at the time of harvest of between 40-80%, and similar densities between experimental and control cells. Cells were collected by trypsin at the times indicated and concentrated by centrifugation. Samples were prepared by resuspending cells in water and boiling for 5 minutes, followed by centrifugation (15 min at 14,000x*g*) to pellet cell debris. Supernatant (S14; 10 µL) was added to a tube containing 100 µL reconstituted luciferin/luciferase reagent, and ATP levels were determined by luminometer, and quantified using an ATP standard curve (10^-7^–10^-10^ M). Reactions were carried out in triplicates (3/well) and averaged. Protein concentrations were determined for the same samples to allow ATP levels to be normalized to cell protein. ATP concentrations were expressed as the percentage of controls as the absolute values differed between experiments. All values are reported as mean ±1 standard deviation.

### Blue Native Polyacrylamide Gel Electrophoresis (BN-PAGE)

4–16% BN-PAGE gels were purchased (Invitrogen) and electrophoresis was carried out according to manufacturer's instructions. Molecular weight standards for native gels were purchased from Invitrogen (cat#LC0725).

### Expression and purification of recombinant proteins

ARL2 and BART-His were expressed from pET3C plasmids. Plasmids were transformed into BL21(DE3) cells and protein expression was achieved by growth in Studier's auto-induction medium [36], as described earlier for wild type ARL2 [18], [37]. Bovine ANT1 was purified as inclusion bodies from *E. coli* as described previously, except that the host cells were *E. coli* C0214(DE3) [38], [39]. Inclusion bodies were purified on a sucrose density gradient, and ANT1 was purified by centrifugation and washing [39] prior to solubilization in Sarkosyl and reconstitution into proteoliposomes.

### ADP/ATP transport assays

The recombinant ANT1 (10 µg) in 1.7% Sarkosyl was reconstituted into liposomes in the presence of 10 mM ADP, 5 mM MgCl_2_ and 10 mM PIPES pH 7.0, as described previously [38],[40]. External substrate was removed from proteoliposomes on Sephadex G-75 columns pre-equilibrated with 50 mM NaCl and 10 mM PIPES, pH 7.0. The amount of ANT1 incorporated into liposomes was measured as described previously [41]; in all experiments, it was ∼20%. Purified recombinant ARL2 or ARL2 and BART-His (30 µg each) were pre-incubated with 5 mM MgCl_2_, and 20 µM GTP or GDP for 20 min, prior to addition to the proteoliposome mixture. Transport at 25°C was started by adding [^14^C]-ADP (Perkin Elmer) to substrate-loaded proteoliposomes (exchange), and terminated after 1 minute by addition of 20 mM pyridoxal 5′-phosphate and 20 mM bathophenanthroline according to the ‘inhibitor stop’ method [42]. The external substrate was removed, and the radioactivity in the liposomes was measured [40]. The initial transport rate was calculated from the radioactivity taken up by proteoliposomes after 1 min (in the initial linear range of substrate uptake). All values are reported as mean ±1 standard deviation.

## Results

**Figure 1:**
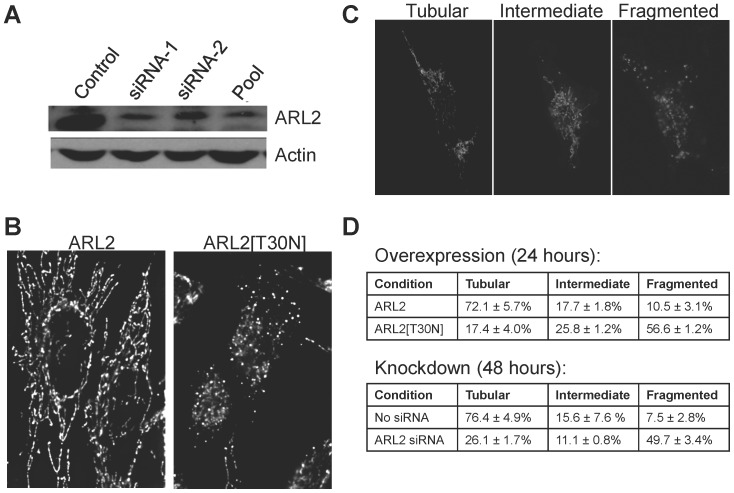
Mitochondria are fragmented in cells where ARL2 activity is compromised. (**A**) HeLa cells were transfected with either no siRNA (control), each of two individual, sequence-independent siRNAs (siRNA-1 and siRNA-2), or a pool of four siRNAs (siRNA pool) and harvested 48 hours after transfection. Equal amounts (25 µg) of total cellular homogenates were resolved in denaturing polyacrylamide gels. The immunoblot was probed with rabbit polyclonal antibody to ARL2 (upper panel) or actin (lower panel). Only the relevant regions of the gels are shown. (**B**) HeLa cells were transfected with plasmids directing expression of either ARL2 (left), or ARL2[T30N] (right), and stained for cytochrome c, as described under Materials and Methods. Representative widefield images with deconvolution of z-stacks are shown. **(C)** HeLa cells were fixed and stained with an HSP60 antibody and data captured using confocal microscopy. Examples of cells from a mock transfected cell population showing tubular (mostly tubular mitochondria), intermediate (mix of tubular and spherical mitochondria), and fragmented (almost all spherical) mitochondria are shown. (**D**) HeLa cells were co-transfected with mito-GFP and either ARL2 or ARL2[T30N] (for overexpression, scored at 24 hours), or mito-GFP plus ARL2 SmartPool or no siRNA (for knockdown, scored at 48 hours). Mito-GFP expressing cells were scored using the criteria in C, and are expressed as percentages of the transfected cell population. At least 200 cells per condition in three independent experiments were scored. Differences between control and experimental were significant to p<0.01 in the tubular and fragmented categories for both ARL2[T30N] and ARL2 siRNA.

To explore the functional role of ARL2 in mitochondria, we initially used two separate approaches to deplete cells of ARL2 activity: knockdown by siRNA and expression of a dominant negative mutant. In the first of these we tested multiple, sequence-independent synthetic siRNAs and plasmid-based shRNAs; including two Dharmacon pools of synthetic RNAs and eight pSUPER-based plasmids [22] to direct expression of shRNAs to knockdown ARL2. The two most effective reagents at reducing ARL2 protein levels (Figure 1A) proved to be commercially available, synthetic RNAs (siRNA #1 and #2) from the SmartPool (Dharmacon) of four siRNAs. Controls for specificity of knockdown include the sequence independence of the RNAs, mock transfected cells, and use of siRNAs directed against the closest ARL2 paralog, ARL3, which lack each of the activities described here for ARL2. ARL2[T30N] is a previously described dominant negative mutant that has decreased affinity for guanine nucleotides [26] and is thus predicted to bind ARL2 GEF(s) and prevent their acting on endogenous ARL2. The key control for effects of ARL2[T30N] expression is comparison to wild type ARL2 over-expression, which consistently is found at higher levels than ARL2[T30N] in cell homogenates, assessed by immunoblotting with ARL2 specific antisera. We believe this is the result of a shorter half-life of the mutant, secondary to its impaired guanine nucleotide binding [26] but have not tested this conclusion.

### Loss of ARL2 activity causes mitochondrial fragmentation

We found that decreasing ARL2 activity in cells using a couple different strategies resulted in fragmentation of mitochondria. HeLa cells were co-transfected with plasmids directing expression of mito-GFP with either wild type ARL2 or ARL2[T30N], and mitochondrial morphologies were observed 24 hours later, as described under Materials and Methods. HeLa cells overexpressing ARL2 and mito-GFP displayed no differences in mitochondrial morphology. In contrast, we found that expression of ARL2[T30N] dramatically altered mitochondrial morphology, leading to fragmented structures that were evident by standard epifluorescence imaging. Even more striking were the deconvolved images of cytochrome c staining in z stacks of control HeLa cells or those expressing ARL2[T30N], as shown in Figure 1B. Extended, branching tubular mitochondrial structures are evident and common in control cells. These are lost in cells expressing ARL2[T30N] and replaced by much smaller structures that appear spherical, despite the fact that this mutant is expressed to lower levels than wild type ARL2. We observe the same phenotype when ARL2[T30N] is expressed in other cell lines, including C2C12, COS7, and MCF7, so we believe these results are applicable to different cell types.

Like expression of ARL2[T30N], knockdown of ARL2 by siRNA also caused mitochondria to fragment. Mitochondrial morphologies in cells with compromised ARL2 activity were similar whether cells were stained with antibodies to TOM20 (marker of the outer membrane), cytochrome c (marker of the intermembrane space), or HSP60 (a marker of the mitochondrial matrix). In addition to changes in mitochondrial morphology, we also noted that cells deficient in ARL2 activity, either resulting from depletion by siRNA or expression of ARL2[T30N], appear to be smaller than controls. This size difference was not quantified or explored further in the current study but was reported earlier in related studies [10], [11], [22].

To quantify the effects on mitochondrial morphology we scored mitochondrial networks in transfected cells as having one of three phenotypes: (1) “tubular”  =  long, tubular and branched structures, (2) “intermediate”  =  a mixture of tubular and fragmented phenotypes, and (3) “fragmented”  =  mainly small and round structures with no tubular or branched structures (Figure 1C). The tubular and intermediate phenotypes are commonly observed in control cells, accounting for ∼90% of all cells. In contrast, cells transfected with ARL2[T30N] or ARL2 siRNA displayed fragmentation in ∼50% of cells (Figure 1D). Thus, mitochondrial fragmentation is a readily scored phenotype that accompanies loss of ARL2 activity.

**Figure 2:**
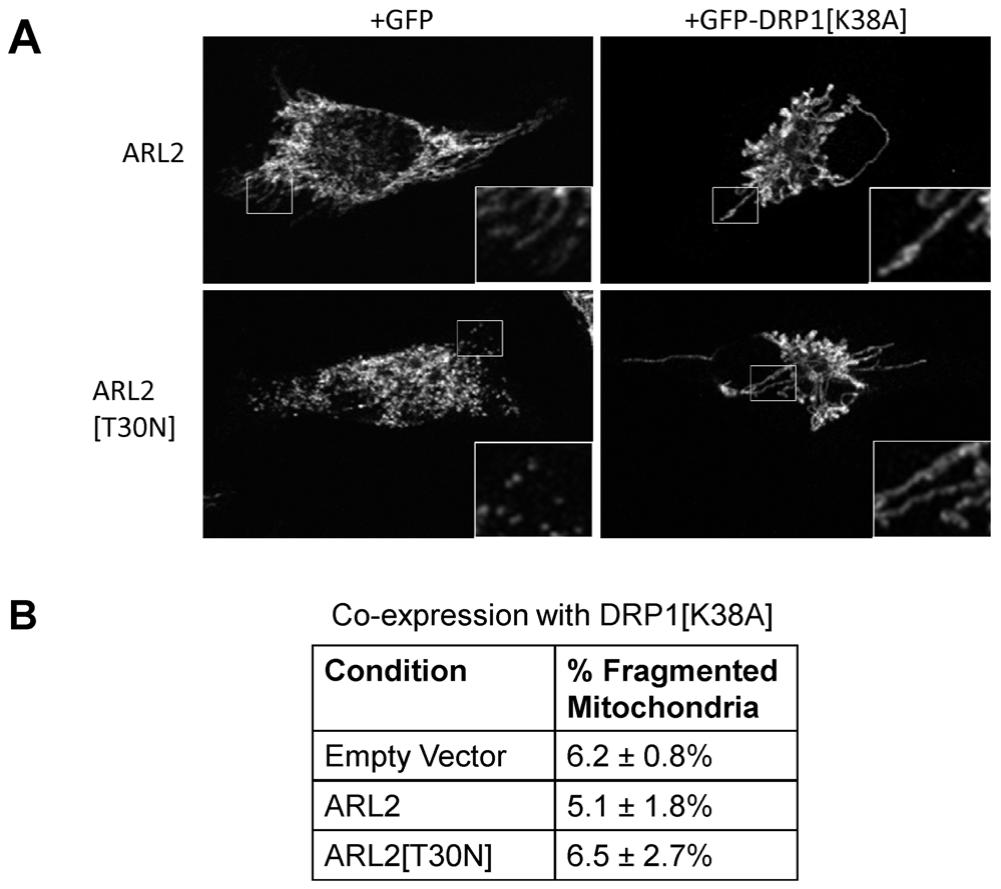
Expression of GFP-DRP1[K38A] reverses fragmentation caused by ARL2[T30N]. HeLa cells were co-transfected with either GFP or GFP-DRP1[K38A] and empty vector, ARL2, or ARL2[T30N]. (**A**) Cells were fixed 24 hours post-transfection and immunostained for HSP60. Representative GFP positive cells are shown. Insets show magnified regions from the cell periphery where the lower densities better highlight mitochondrial morphology. Cells expressing wild type ARL2 have similar mitochondrial morphology to cells transfected with empty vector (not shown). (**B**) Cells expressing GFP-DRP1[K38A] were scored for fragmented mitochondria, using the criteria illustrated in Figure 1D. Cells were quantified from three independent experiments.

Mitochondrial fragmentation can result from either a loss of fusion or increase in fission [43]. To distinguish between these two activities, we used the dominant negative mutant of the fission component DRP1, DRP1[K38A], as previously described [31]. Expression of DRP1[K38A] blocks mitochondrial fission, while fusion proceeds normally, leading to elongated mitochondria. Therefore, mitochondria should only fragment with expression of DRP1[K38A] if fusion is impaired, as DRP1[K38A] should block fission. GFP or GFP-DRP1[K38A] was co-expressed with empty vector or those expressing ARL2 or ARL2[T30N], and mitochondrial morphologies in GFP positive cells were scored. Expression of DRP1[K38A] resulted in mitochondria with increased apparent length and interconnectivity when co-expressed with either empty vector (not shown) or wild-type ARL2 (Figure 2A, top right panel), compared to cells co-expressing GFP and pcDNA (not shown) or wild-type ARL2 (Figure 2A, top left panel). Co-expression of GFP-DRP1[K38A] reversed mitochondrial fragmentation caused by ARL2[T30N] (Figure 2A, bottom right panel), despite the fact that ARL2[T30N] caused mitochondrial fragmentation when co-transfected with GFP alone (Figure 2A, bottom left panel).Cells co-transfected with GFP-DRP1[K38A] and empty vector, ARL2 wild type, or ARL2[T30N] were scored for fragmented mitochondria, and we observed no statistically significant differences (Figure 2B). Both ARL2 and ARL2[T30N] expressed to similar levels, regardless of co-expression with GFP alone or GFP-DRP1[K38A] (Figure S1). Because DRP1[K38A] blocks mitochondrial fission, we interpret these findings as indirect evidence that a loss in ARL2 activity alters mitochondrial morphology by increasing fission at a step that is upstream of the actions of DRP1. Stated another way, we believe that ARL2 may act to down-regulate fission such that its loss results in increased fission.

### Mitochondrial motility is specifically compromised in cells deficient in ARL2 activity

**Figure 3:**
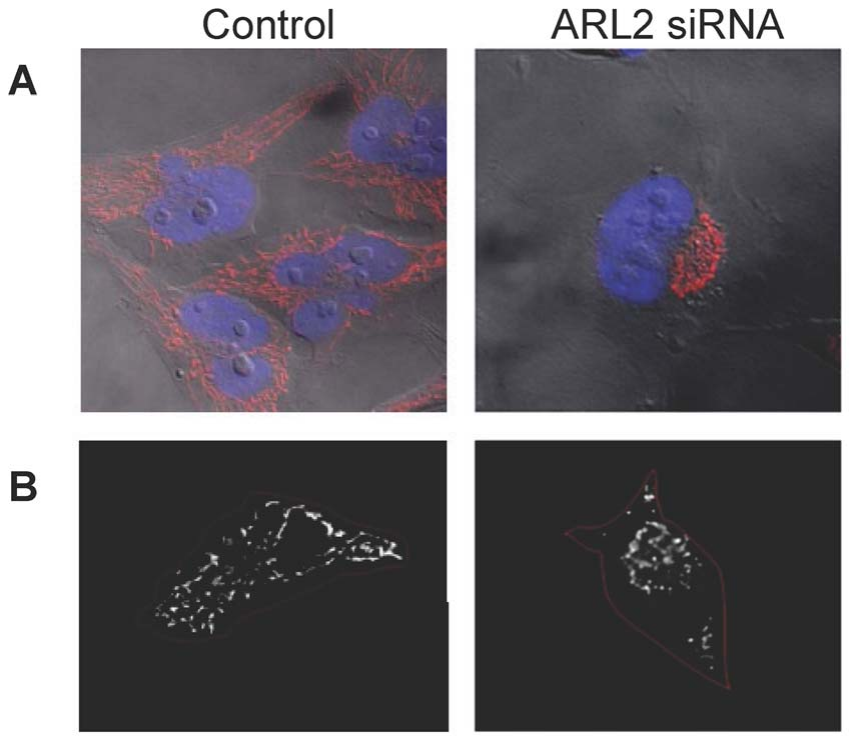
Mitochondria cluster in the perinuclear region in cells depleted of ARL2. (**A**) HeLa cells were either mock transfected (Control, left panels in A and B), or transfected with the SmartPool of ARL2 siRNAs (right panels in A and B), and the next day were fixed and stained for cytochrome c, as described under Materials and Methods. Cytochrome c staining is shown in red and overlaid onto phase contrast images, along with nuclear (Hoechst) stain, shown in blue. Representative cells are shown. (**B**) Cytochrome c staining is shown in white, with the cell borders outlined in red to highlight the perinuclear clustering in cells lacking ARL2 activity.

In addition to the fragmentation of mitochondria there was an increase in perinuclear clustering of mitochondria in cells depleted for ARL2 (Figure 3A,B) or expressing ARL2[T30N]. This clustering was most readily seen as an absence, or clearly diminished density, of mitochondria in the cell periphery as mitochondria in control cells typically appear to have a higher density near the nucleus for a number of reasons (e.g., thickness of the cell). This perinuclear clustering with loss of peripheral mitochondria was almost completely absent in control cells (0.9%) but was seen in 23.7±0.5% of cells depleted of ARL2.

**Figure 4:**
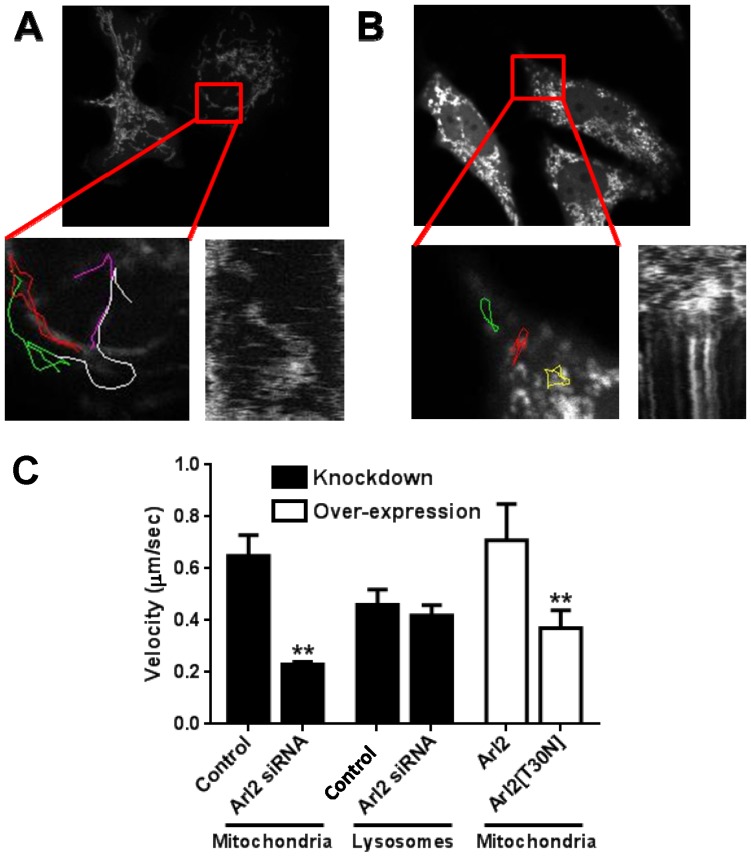
Loss of ARL2 activity compromises mitochondrial motility. HeLa cells were transfected with plasmids directing expression of mito-GFP or mito-GFP plus ARL2 siRNAs. After 24 hours cells displaying normal appearing mitochondria were imaged over time, as described in Materials and Methods. Examples of control (**A**) and knockdown (**B**) cells are shown, with mito-GFP fluorescence in the top panels, blow ups of the boxed areas bottom left, and kymographs from that region shown as a function of time below right. Colored lines showing tracking of individual mitochondria are shown in the bottom left of each panel with different colors indicating different particles tracked. (**C**) Velocities of organelle movements were determined as described under Materials and Methods and averaged over the entire eight hour recording period. HeLa cells were depleted of ARL2 by siRNA (solid bars) or transfected to over-express wild type (ARL2) or the dominant negative mutant (ARL2[T30N] (open bars)). Lysosome movements were recorded with time lapse imaging in control or ARL2 siRNA cells by incubating cells with LysoTracker Red 30 minutes prior to imaging. Double asterisks indicate statistically significant differences (p<0.01).

In efforts to gain further insights into possible relationships between fragmentation and clustering of mitochondria we performed time-lapse imaging. Control cells, expressing only mito-GFP, were characterized by mitochondria moving in a bidirectional fashion with thread-like morphology that displayed readily observable fusion and fission events. Substantial mitochondrial fragmentation, perinuclear clustering, or sustained loss of motility were rare in controls. In contrast, cells depleted of ARL2 by siRNA displayed mitochondrial fragmentation (52.3±6.5%), with or without clustering, by the end of the eight hour imaging window. Perinuclear clustering of mitochondria occurred in 16±3.1% of imaged cells, resulting from directed movement of the fragmented mitochondria inward, with a near complete absence of outward movement. And in a similar percentage of cells (14.1±4.6%), we observed loss of mitochondrial motility, with fragmented mitochondria simply stopping their directed movement throughout the cell but retaining localized movement. The remaining (∼70%) transfected cells displayed bidirectional, motile, fragmented mitochondria. Thus, perinuclear clustering resulted from a loss in outward mitochondrial motility and occurred after mitochondrial fragmentation. Kymographs of mitochondria in time lapse images from mito-GFP expressing HeLa cells were produced using ImageJ, as described previously [35]. Motile mitochondria were identified as moving objects in movies and diagonal lines in the kymographs (Figure 4A,B). Mean velocity of motile mitochondria was quantified using ImageJ to track individual mitochondria and dividing displacement distance by time (Figure 4C). Rates of mitochondrial motility in ARL2 siRNA cells (0.23±0.01 µm/sec; 28 mitochondria from 8 cells) were less than half those in control cells (0.65±0.08 µm/sec; 51 mitochondria from 8 cells) when averaged over the eight hour imaging window (Figure 4C). These control rates from this experiment of mitochondrial transport are very similar to previously reported values [35], [44], [45].

We also observed changes in mitochondrial motility in cells expressing ARL2[T30N]. HeLa cells expressing mito-GFP or mito-GFP and ARL2 were imaged as described above. No differences in the overall distribution, length, or motility of mitochondria were evident by visual inspection, and there was no significant difference between the average rate of movement in controls (0.77±0.12 µm/sec) and ARL2-expressing cells (0.71±0.14 µm/sec). In contrast to controls, but similar to cells depleted for ARL2, cells expressing ARL2[T30N] displayed mitochondria that were fragmented and arrested in their motility. The mean velocity of mitochondria in ARL2[T30N] expressing cells was 0.37±0.7 µm/second, a 48% decrease (p<0.01) from ARL2 over-expressing cells (Figure 4C). Together these data reveal that loss of ARL2 activity, through either ARL2 depletion or expression of ARL2[T30N], causes a significant decrease in the average rate of mitochondrial movements.

Transport of other organelles, e.g., lysosomes and endosomes, also occurs along microtubules but these were not affected by depletion of ARL2. The rate of movement of lysosomes (determined as described in Materials and Methods) was found to be the same in control (0.46±0.06 µm/sec; average of five cells/experiment, repeated three times) and ARL2 siRNA cells (0.42±0.04 µm/sec) (Figure 4C) and was comparable to previously reported values [46]. The movements of early and late endosomes (visualized by GFP-RAB5 or RAB7, respectively) over the imaging period were indistinguishable between control and ARL2 siRNA cells upon visual inspection, so we did not quantify further. To further ensure that mitochondria movements were compromised under conditions in which lysosome and endosome mobilities were preserved, we also performed experiments in which we tracked both mitochondria and lysosomes in the same cells; comparing controls to either ARL2 depletion or ARL2[T30N] expression. In each case we observed instances in which mitochondrial motility was slowed by the loss of ARL2 activity (either ARL2 siRNA or ARL2[T30N]), yet lysosomal and endosomal traffic continued without evident change. Thus, depletion of ARL2 resulted in a motility defect that was specific to mitochondria and was not shared by other organelles, which traffic along similar cytoskeletal elements.

### Mitochondrial phenotypes from loss of ARL2 activity are not secondary to changes in microtubules

Previous work in our lab had described effects of the dominant *active* mutant ARL2[Q70L] to cause the loss of microtubules [22]. In addition, work by our lab and others describes a role for ARL2 in regulation of microtubule destruction through the tubulin co-chaperone cofactor D [2], [3], [47], [48]. Given ARL2's well-established role as a regulator of tubulin folding and microtubule dynamics [2], [3], [4], [5], [6], [7], [8], [9], we wanted to clearly distinguish between direct effects of the GTPase on mitochondria as opposed to possible indirect effects resulting from changes in microtubules.

In an unrelated study we had generated a point mutant that helped us to more clearly resolve these two functions of ARL2, a conservative point mutant of ARL2, Lys71-Arg71 or ARL2[K71R]. We expressed ARL2[K71R] in HeLa cells, using the same pcDNA3-based vectors used above for wild type and ARL2[T30N], and assayed effects on mitochondria. ARL2[K71R] also caused mitochondria to fragment and cluster around the nucleus when assessed 24 or 48 hours after transfection. Thus, by these criteria ARL2[K71R] appears to act in a dominant negative fashion, indistinguishably from ARL2[T30N] or ARL2 siRNA. ARL2[K71R] was expressed to the same levels as ARL2, each of which are higher than the T30N mutant, as determined by immunoblotting of whole cell lysates. Thus, ARL2[K71R] phenocopies ARL2[T30N] and ARL2 siRNA in promoting mitochondrial fragmentation and perinuclear clustering.

**Figure 5:**
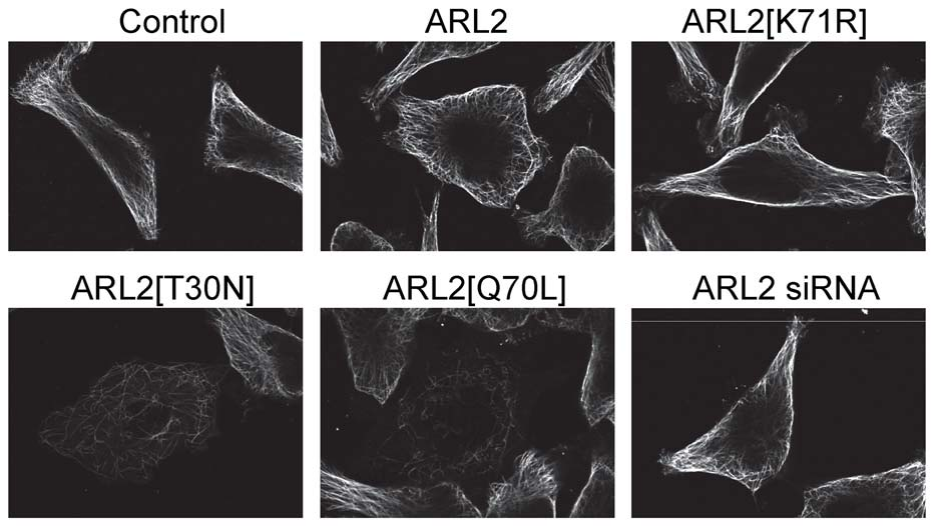
Microtubules are not lost with expression of ARL2[K71R] or ARL2 siRNA. HeLa cells were transfected with plasmids directing expression of ARL2, ARL2[T30N], ARL2[K71R], ARL2[Q70L], or with ARL2 siRNA #1. Cells were fixed 48 hours later and stained for α-tubulin, as described under Materials and Methods. Representative confocal images are shown, though the extent of microtubule density loss with ARL2[T30N] and ARL2[Q70L] vary and is more severe with the latter.

When we compared the microtubule staining profiles of mock transfected cells with those expressing ARL2 or ARL2[K71R], and those depleted for ARL2, we found that microtubule profiles were indistinguishable at all times examined (Figure 5; 48 hours); characterized by meshwork staining throughout the cytosol, extending to the cell periphery. In contrast, only cells expressing ARL2[T30N] or ARL2[Q70L] displayed a clearly reduced density of microtubules (Figure 5). This effect on microtubules was seen 48 hours after transfection (Figure 5), which is later than when we observe mitochondrial defects (24 hours). The strongest effect on loss of microtubules was seen with expression of the dominant active mutant, ARL2[Q70L] (Figure 5) [22], which had no effects on mitochondrial fragmentation or clustering (data not shown).

Thus, the two point mutants ([T30N] and [K71R]) whose expression cause very similar changes to mitochondrial morphology and clustering have very different effects on microtubules. Additionally, knockdown of ARL2 by siRNA did not alter microtubule density. Lastly, we showed that endosomal and lysosomal motility are not compromised at the same times that we observe mitochondrial motility loss. Taken together (see summary in Table 1), these results strengthen our conclusions that ARL2 regulates multiple aspects of mitochondrial function and does so independently of its role(s) in tubulin or microtubule dynamics.

**Table 1:** Summary of phenotypes resulting from over-expression of ARL2, expression of ARL2 mutants, or depletion of ARL2 or its binding partners.

Condition	Fragmentation	Perinuclear Clustering	ATP Loss	Microtubule Loss	Time point
ARL2	−	−	−	−	24, 48 hours
ARL2 siRNA	+	+	+	−	24 hours (fragmentation and clustering), 48 hours (ATP loss)
ARL2[T30N]	+	+	−	+	24 hours (fragmentation and clustering), 48 hours (microtubule loss)
ARL2[K71R]	+	+	−	−	24, 48 hours
ARL2[Q70L]	−	−	N.D.	+	48 hours
ELMOD2 siRNA	+	+	−	−	48 hours
BART siRNA	−	−	−	N.D.	24, 48 hours

Conditions and brief descriptions of the effects are shown on the left. (+), an effect was observed, as described in the text; (−) no effect; N.D., not determined.

### ARL2 siRNA, but not ARL2[T30N], causes loss of cellular ATP

**Figure 6:**
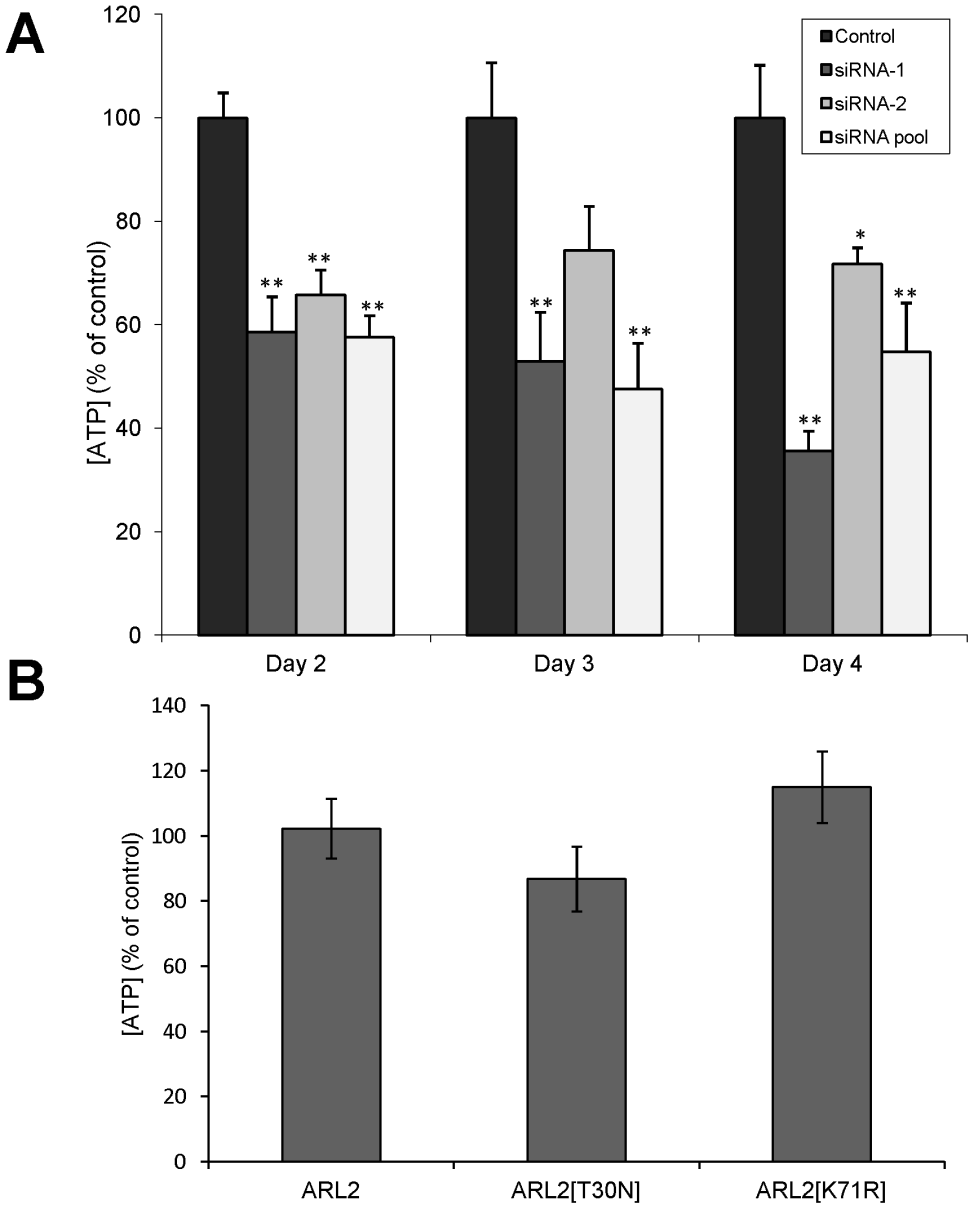
ATP levels are lower in cells depleted of ARL2 by siRNA. (**A**) HeLa cells were transfected with either no siRNA (control), each of two individual siRNAs (siRNA-1 and siRNA-2), or a pool of four siRNAs (siRNA pool) and collected 48 hours after transfection for ATP determinations, as described under Materials and Methods. A single or double asterisk indicate statistically significant differences at p<0.05 and p<0.01, respectively. (**B**) HeLa cells were transfected with ARL2, ARL2[T30N], or ARL2[K71R] and assayed for ATP levels as in (A).

Given that loss of ARL2 activity, via siRNA-mediated knockdown or expression of ARL2[T30N], causes changes to mitochondrial morphology and motility, we next asked if loss of ARL2 activity compromised ATP production. Depletion of ARL2 by siRNAs in HeLa cells resulted in a dramatic (40–50% of controls) loss of cellular ATP (Figure 6A) 2–4 days after transfection. There was a good qualitative agreement between effectiveness in knockdown of protein expression, assessed by Western blotting of total cell lysates, and effects on ATP levels, with siRNA #1 slightly better than the pool, and siRNA #2 least effective (compare Figure 1A and Figure 6A). The 40–60% decrease in ATP levels is comparable to what has been reported previously after treatment with potent inhibitors of glycolysis or oxidative phosphorylation. In contrast, we saw no significant changes (93%±19%) in cellular ATP 24 hours after transfection with ARL2 siRNA, despite the fact that mitochondria were already fragmented and clustered at this time point, suggesting that the ATP loss occurs after mitochondrial morphology and motility have already been compromised.

Depletion of cellular ARL2 activities through siRNA resulted in decreased numbers of cells compared to controls. This was thought *not* to be the result of apoptosis or loss of mitochondrial membrane potential as we saw no evidence of release of cytochrome c from the IMS or changes in mitochondrial staining with JC-1 or MitoTracker Red CMXRos, two mitochondrial membrane potential-sensitive dyes. Nor did we observe nuclear blebbing. Differences in cell number between ARL2 knockdown and controls increased with time and may result from a combination of increased doubling times and necrosis as previously reported for other mitochondrial proteins [49], [50], though these were not pursued further.

In contrast to ARL2 siRNA, we found that over-expression of wild type human ARL2 (102.3±9.2%) or expression of the dominant negative mutant, ARL2[T30N] (86.8±9.9%) or ARL2[K71R] (115±11%), had no statistically significant effect on ATP levels in HeLa cells after 48 hours, when compared to empty vector controls (Figure 6B). Therefore, we conclude that the changes in mitochondrial morphology and motility accompanying the loss of ARL2 are not secondary to the loss of ATP in cells. These data also highlight the important functional differences between the absence of ARL2 in cells (knockdown) and the presence of a dominant negative mutant, as they act via distinct mechanisms yet share a subset of phenotypes.

### Changes in ARL3 levels or activity have no effect on mitochondria

ARL3 is the closest paralog to ARL2 in humans, sharing 53% identity and 72% homology. With previous evidence of both shared and distinct functions in cells [22], [24], we tested for effects of ARL3 siRNA or expression of dominant negative ARL3[T31N] on mitochondria. We reported previously the characterization of ARL3 siRNA reagents and their effects on Golgi, microtubules, and cytokinesis but did not specifically examine mitochondria in the earlier study [22]. When ARL3 was depleted using an ARL3 SmartPool (Dharmacon) in HeLa cells, we observed no changes in mitochondrial morphology or localization after staining of fixed cells with HSP60 or cytochrome c 48 hours after transfection (data not shown). Similarly, expression of either wild type ARL3 or the dominant negative ARL3[T31N] had no evident effects on mitochondrial morphology or localization in cells. As the distribution of mitochondria was indistinguishable from controls, we did not attempt to quantify mitochondrial motility in either case. We assayed total cellular ATP levels in mock or ARL3 knockdown cells and found no statistically significant differences. Finally, staining of HeLa cells using fixation and permeabilization conditions that are positive for ARL2 in mitochondria failed to reveal any evidence for a pool of ARL3 associated with mitochondria, using our ARL3-specific antisera that is useful in localization to other sites [22]. Thus, we conclude that ARL3 does not share with ARL2 any of the activities described here with regard to mitochondrial functions. Rather, these results further highlight the specificity of these effects to ARL2.

### ARL2 is present in the mitochondrial matrix

Studies in our lab demonstrated that ARL2 from different cells (HeLa, sf295) or tissues (brain, liver, kidney, spleen, heart) partially localizes to mitochondria, as visualized by indirect immunofluorescence microscopy with specific antibodies and by cell fractionation [13]. In that fractionation study, the mitochondria-associated ARL2 was shown to be resistant to protease treatment, unless detergent was added to solubilize the mitochondrial membranes, revealing there to be a pool of ARL2 inside the organelle. Results from sub-mitochondrial fractionation were interpreted as evidence that ARL2 was present in the intermembrane space (IMS) [51]. With evidence for a role for ARL2 in three mitochondrial phenotypes, we re-opened the question of localization within mitochondria using independent methods to determine the localization of ARL2 inside mitochondria.

We used cBid-induced permeabilization of the outer membrane [52] of cultured HeLa cells to test whether ARL2 exists in the IMS in a freely diffusible form. Addition of purified cBid (10 nM; a kind gift of Dr. David Andrews, McMaster University, Canada) to HeLa cells in which the plasma membrane had been permeabilized with 0.2% saponin resulted in essentially complete release of cytochrome c from mitochondria within 15 minutes, as visualized by staining of fixed cells (Figure S2). In contrast, ARL2 staining in mitochondria was retained in HeLa cells treated with cBid under these same conditions (Figure S2). We conclude from these results that ARL2 is unlikely to be free in the IMS and more likely is either bound tightly to another protein(s) on the inner or outer mitochondrial membranes or resides in the matrix of the mitochondria.

**Figure 7:**
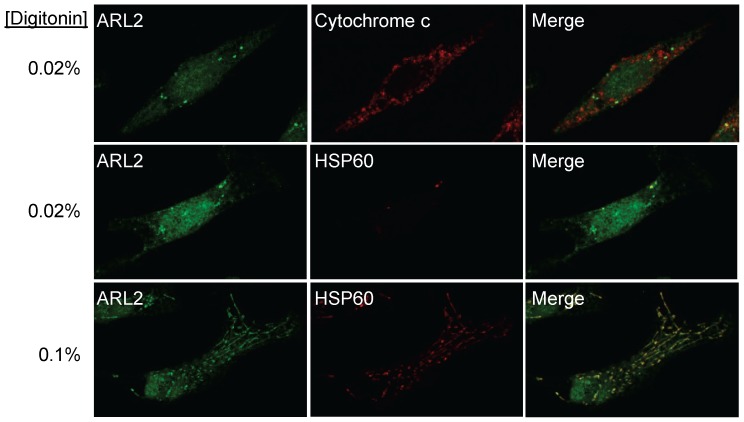
A pool of ARL2 localizes to the mitochondrial matrix. HeLa cells were fixed in 4% paraformaldehyde prior to permeabilization in either 0.02% (two upper rows) or 0.1% w/v digitonin (bottom row) for 10 minutes at room temperature. Cells were then processed for imaging using dual labeling for ARL2 (green, left) and either cytochrome c (top row, middle panel) or HSP60 (lower two rows, middle panels), as markers of the IMS and matrix, respectively. Merged images are shown on the far right in each case.

We then used increasing concentrations of digitonin on fixed cells and looked for the appearance of staining for cytochrome c (as a marker of the IMS), or HSP60 (as a marker of the matrix). Treatment with 0.02% digitonin permeabilized the plasma membrane and outer mitochondrial membrane, as revealed by the appearance of cytochrome c staining (Figure 7, top row). However, no mitochondrial staining for ARL2 or HSP60 was evident (top two rows) under this condition, consistent with the lack of permeabilization of the inner mitochondrial membrane. In contrast, treatment with 0.06–0.1% digitonin led to the emergence of staining for both ARL2 and HSP60 (Figure 7, bottom row). Intermediate concentrations of digitonin were also tested and in every case we found a strong correlation between the appearance of ARL2 and HSP60 staining (data not shown). Note that digitonin also permeabilized the nuclear envelope, revealing ARL2 staining within the nucleus, as previously reported [22]; however, the mitochondrial staining is readily distinguished from the nuclear pool (Figure 7, bottom panels). While these data cannot exclude the presence of some ARL2 in other compartments, including the IMS, we conclude that there exists a mitochondrial pool of ARL2 in the matrix.

### Neither ANT1 nor BART mediate the mitochondrial effects of ARL2

The direct binding of the ARL2(GTP)-BART complex to ANT1 in our gel overlay assay [13] identified the ADP-ATP transporter ANT1 as a potential mediator of the ARL2 effects described above. A prediction from those data was that ARL2 positively regulates ANT transporter activity, and that the absence of ARL2(GTP)-BART binding to ANT causes a decrease in ATP/ADP transport, leading to an accumulation of ADP in the cytosol and falling ATP levels. In support of this idea, another group showed that BART knockdown lowered ATP levels and that ARL2 knockdown led to a decrease in ANT transporter activity in isolated mitochondria [14]. To test this model we used a reconstitution assay for transport proteins, as described previously [40]. The bovine ADP/ATP carrier, isoform 1 (ANT1 or AAC1/SLC25A4), was reconstituted into liposomes, as described under Materials and Methods, and the rate of adenine nucleotide exchange was measured. No differences in the rate of transport were seen upon inclusion of up to 30 µg ARL2 or BART in the reconstitution assay. Using external and internal ADP concentrations of 0.02 and 10 mM, respectively, the transport activity of reconstituted ANT1 was 73.2±8.1 µmol/min/g protein in controls (i.e., in the absence of either ARL2 or BART). The activity was no different in the presence of BART (71.5±8.7 µmol/min/g protein), or ARL2 alone (75.0±10.2 µmol/min/g protein) or the ARL2(GTP)-BART complex (72.6±11.2 µmol/min/g protein) or ARL2(GDP)/BART (70.1±9.4 µmol/min/g protein).

Any *in vitro* reconstitution may fail to replicate a response seen in cells as a result of the absence of a required component that is unknown. BART is the only known classical ARL2 effector in mitochondria, and was required for ARL2 binding to ANT1 in our gel overlay assay [13]. Thus, to further test the possibility that the effects of ARL2 siRNA on ATP levels were mediated by loss of ARL2 binding to BART (and therefore ANT1), we performed knockdowns of BART in HeLa cells. Four siRNAs against BART (Dharmacon) each decreased BART levels by an estimated 80%, as judged by immunoblotting of cell homogenates up to four days after transfection. With successful depletion of BART protein from HeLa cells, we looked for effects on mitochondrial morphology and ATP levels in cells. HeLa cells were fixed on days 1–3 after transfection with the siRNAs and stained with an antibody to cytochrome c to image mitochondria. No differences were apparent in overall mitochondrial morphology or localization in cells, as determined by visual inspection. Total cellular pools of ATP in BART siRNA cells were not significantly different from control cells after 48 hours of knockdown (96.5±9.7% of control levels for BART siRNA #1, and 106.6±1.4% for BART siRNA #2). Thus, despite the fact that a pool of BART is found within mitochondria and our previously published data revealed that BART was required for ARL2(GTP) to bind ANT1 in a gel overlay assay, these data fail to support a role for ANT1 or BART in mediating effects of ARL2 on ATP levels, mitochondria morphology, or motility in HeLa cells.

With the lack of evidence in ARL2(GTP)-BART regulation of ANT1, we sought another explanation for the large ATP loss observed with ARL2 siRNA. There is precedent for proteins with well-established roles outside of mitochondria, but with smaller pools inside mitochondria, also having critical roles in maintenance of ATP levels and stability of electron chain complexes (e.g., MEF2 [53] and STAT3 [54]). Thus, we tested for changes in the abundance of complexes I-V in mitochondria after depletion of ARL2. We monitored the levels of each complex using non-denaturing blue native-polyacrylamide gels to resolve the complexes and antibodies to a component of each complex (see Materials and Methods for details) to estimate the abundance of each and found no differences of any of these five complexes in ARL2 siRNA cells compared to mock transfected controls.

### Knockdown of the ARL2 GAP, ELMOD2, phenocopies ARL2 point mutants

**Figure 8:**
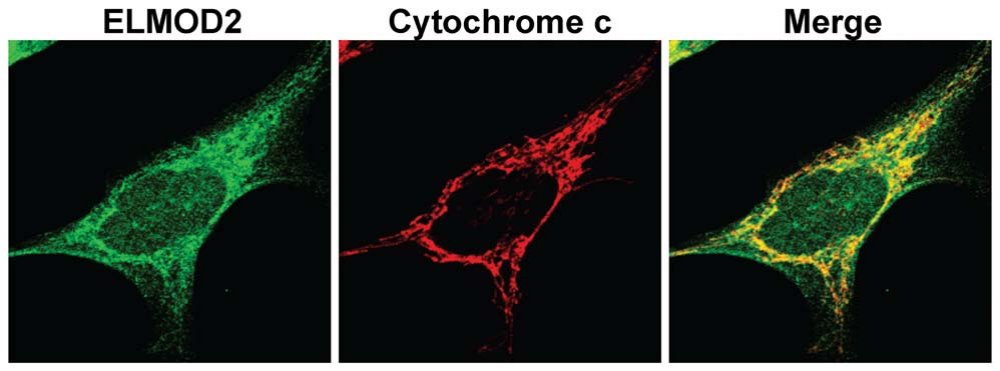
ELMOD2 localizes to mitochondria. HeLa cells were fixed in 4% paraformaldehyde, permeabilized in 0.1% Triton X-100 and stained for ELMOD2 (green, left) and cytochrome c (red, middle). ELMOD2 staining overlaps extensively with that of cytochrome c (Merge, right).

We next explored the possibility that one or more of the three recently identified ARL2 GAPs, ELMOD1-3 [18], [19], [20], may localize to mitochondria and act downstream of ARL2. Little is known of the functions of these three proteins in cells and GAP activity is the only known biochemical activity for any of them [19], [55]. Because ARF family GAPs are often found to have effector functions [56], we explored the potential for ELMOD proteins as effectors for ARL2 in mitochondria. We developed a rabbit polyclonal antibody to human ELMOD2 and found specific localization of endogenous protein to mitochondria in HeLa (Figure 8) and other cells (e.g., COS7, SH-SY5Y; Figure S3). The specificity of our rabbit polyclonal antibody to ELMOD2 and mitochondrial staining was confirmed in control studies testing pre-immune serum and antigen competition on fixed cells (Figure S3). Specific localization to mitochondria was further confirmed by expression in HeLa cells of epitope (HA) tagged ELMOD2-HA as antibodies to the epitope tag overlapped extensively with mitochondrial markers, cytochrome c or HSP60 (data not shown). Using the same method described above of increasing concentrations of digitonin to solubilize different mitochondrial membranes we found that at least some of this mitochondria localized ELMOD2 is present in the mitochondrial matrix (Figure S4).

**Figure 9:**
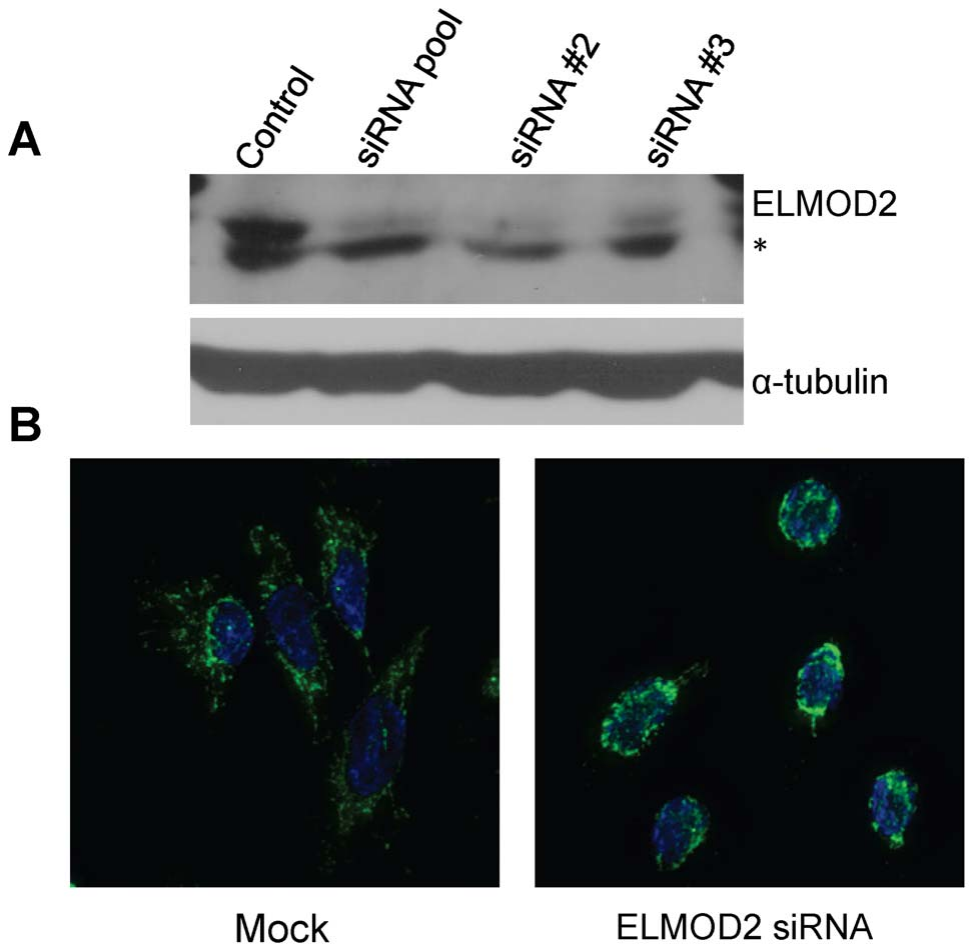
ELMOD2 knockdown alters mitochondrial morphology and distribution. (**A**) HeLa cells were transfected with a SmartPool or individual siRNAs directed against ELMOD2 and two days later cells were harvested, total protein determined, and equal amounts (40 µg) of total cellular homogenates were resolved in denaturing polyacrylamide gels. Proteins were transferred to nitrocellulose membranes and probed with our rabbit polyclonal antibody to ELMOD2 (upper panel) or α-tubulin (lower panel). Only the relevant regions of the gels are shown. Asterisk indicates a non-specific band, migrating slightly faster than ELMOD2, which is not competable with antigen. (**B**) HeLa cells were transfected with either no siRNA (Mock) or siRNA directed against ELMOD2 and two days later cells were fixed and stained with Mitotracker and Hoechst DNA stain. Representative cells are shown. Note the proximity of mitochondrial staining to the nuclear stain and the lack of peripheral mitochondrial staining in ELMOD2 knockdown cells.

We next tested commercial siRNA reagents to allow us to evaluate a functional role for ELMOD2 in mitochondria. Dharmacon's Smart Pool of four siRNAs as well as two individual siRNAs purchased from Sigma Chemical Co were found to effectively deplete ELMOD2 protein, as assayed by immunoblot. Each of these siRNAs effectively depleted cells of ELMOD2 on days 2 (Figure 9A) or 3 (data not shown). When cells were fixed at these time points and imaged with mitochondrial markers we found them to be fragmented and concentrated around the nucleus (Figure 9B) similar to phenotypes described above for ARL2 knockdown. When assayed for total cellular ATP levels at 48 hours we found no significant differences between controls and ELMOD2 siRNA (98.4±7.0% for siRNA #2, 98.7±10% for siRNA #3). These data are consistent with a model in which ELMOD2 acts downstream of ARL2 in regulating mitochondrial morphology and motility, but not in its actions required for maintenance of ATP pools.

## Discussion

We provide evidence that ARL2 acts in mitochondria to regulate mitochondrial morphology, motility, and ATP production. Differences between the consequences of ARL2 siRNA compared to expression of two different dominant acting point mutants allowed us to cleanly resolve effects on ATP levels or microtubules from those on mitochondria morphology or motility (summarized in Table 1). Thus, ARL2 has been shown to have a distinct and essential role in mitochondria that expands its known roles in eukaryotic cell biology. These roles at least provide an opportunity for coordinate regulation of energy metabolism and the cytoskeleton, with likely links to cell division. Together, our data lead us to propose the existence of two actions of ARL2 relevant to mitochondrial physiology that are each distinct from effects on tubulin or microtubules; one impacting inner membrane ultrastructure with consequences to ATP production and one involving ELMOD2 that results in changes in morphology and motility. These models are discussed in more detail below.

The effects of ARL2 siRNAs on ATP pools are consistent with the previously described [14], [57] effects of microRNAs (miRNAs) that target ARL2 and lead us to conclude both that ARL2 is required for maintenance of ATP levels and that at least some tissues have the means to modulate ARL2 levels post-transcriptionally. Four or more microRNAs may target ARL2 mRNA in cardiac tissues [14], [57], and depletion of ARL2 in neonatal ventricular myocytes also lowered ATP levels [14]. Earlier, we found that levels of *mitochondrial* ARL2 (no changes in total tissue ARL2) increased several fold in mice deleted for the adenine nucleotide transporter, ANT1, but only in cardiac and skeletal muscle [13], indicating the existence of regulated import of ARL2 into mitochondria that may be tissue specific. With (i) the levels of cellular ARL2 under regulation by miRNAs; (ii) the regulated import of ARL2 into mitochondria, via unknown mechanisms; and (iii) the ancient and highly conserved nature of ARL2 and its ubiquitous expression, we believe that ARL2 plays fundamental roles in energy metabolism in mitochondria of all eukaryotic cells, though ARL2 function may be particularly important in those cells with high energy demands, e.g., cardiac and skeletal muscle cells. This requirement for ARL2 in the maintenance of ATP pools may explain its essential nature, previously described in a number of organisms, though in those studies clear resolution from effects on microtubules and cell division was not achieved.

In addition to effects on ATP levels, changes in ARL2 activity cause fragmentation of mitochondria and specific loss of mitochondrial motility. We interpret the observation that fragmentation of mitochondria was prevented in cells expressing both ARL2[T30N] and DRP1[K38A], which inhibits mitochondrial fission, as evidence that the ARL2 mutant alone promotes mitochondrial fission, a process that is thought to require attachment to mitochondria and motility [58], and that ARL2 acts upstream of DRP1 to do so. Miro is a calcium-sensitive GTPase [58], [59] that is involved in mitochondrial motility and ELMOD2 is a GAP for ARL2 with broad specificity, so far restricted to the ARF family, but may be worth exploring [18] for possible functional connections. These effects were clearly resolved from changes in ATP levels as they were evident in cells expressing ARL2[T30N] or ARL2[K71R], which do not alter ATP levels, and thus are not simply secondary consequences of falling ATP. In addition, substantial changes in morphology and motility were evident within 24 hours, at which point we saw no statistically significant changes in ATP levels. We believe the order of events resulting from loss of ARL2 is fragmentation, loss of motility, and then loss of ATP.

Because changes in ARL2 activity (e.g., expression of the dominant active mutant ARL2[Q70L] or ARL2[T30N]) can also result in changes in microtubule density in cells and mitochondria travel along microtubules, it was important to cleanly resolve these two activities. The result that depletion of ARL2 (siRNA) or expression of ARL2[K71R] each cause mitochondrial fragmentation and loss of motility without changes in microtubules is evidence that ARL2 activity is required for maintenance of mitochondrial morphology and motility independently of effects on microtubules. In addition, the fact that endosomes and lysosomes, which also traffic along microtubules, were found to travel at undisturbed rates in the same cells that lost mitochondrial motility is strong evidence of a specific defect at mitochondria and argues further against changes in microtubules or ATP levels as primary defects.

Our conclusion that ARL2 is required for mitochondrial morphology, motility, and ATP production led us to test our previous model, that ARL2 is an activator of ATP/ADP transporters in the inner mitochondrial membrane [13], [14]. Our earlier *in vitro* data demonstrated direct binding of ANT1 by the ARL2(GTP)-BART complex, but no functional evidence of effects on transporter activities. Here we used reconstitution assays of ANT1 in proteoliposomes and failed to see any changes in transporter activity upon addition of ARL2, BART, or the ARL2(GTP)-BART complex. We also found that depletion of BART had no effects on ATP levels or mitochondrial morphology in the same cell line in which depletion of ARL2 did. This is in contrast to Nishi, et al [14], who reported that BART siRNA lowered ATP levels, though we cannot currently explain the different outcomes. Thus, we found no evidence that BART is a mediator of the required roles of ARL2 in maintenance of ATP levels, mitochondria fragmentation, or motility, despite its presence in the matrix. Though our results do not rule out other, currently unknown, roles for ANT and BART in ARL2 signaling, we conclude that our mitochondrial phenotypes resulting from the loss of ARL2 are not mediated by either ANT1 or BART.

In contrast to BART, the evidence that ELMOD2 siRNA phenocopied two of three effects described for ARL2 siRNA, and that both proteins are present in the matrix, is consistent with ELMOD2 being an effector of ARL2 in mitochondria; potentially in regulating morphology and motility but not ATP levels. ELMOD2 is an ARF family GAP with broad substrate specificity within the ARF family [18], [55] and is predicted to act immediately downstream of a GTPase. While some shared binding partners and activities have been previously described for ARL2 and ARL3 [24], [60], [61], we found no evidence of ARL3 localizing to mitochondria or affecting its functions, which suggests a high degree of specificity to ARL2 in its mitochondrial roles and also leaving ELMOD2 as the most likely GAP and effector for at least a subset of ARL2 actions in mitochondria.

We showed that ARL2 is present in the mitochondrial matrix using two different approaches. cBid addition to permeabilized HeLa cells released cytochrome c but retained ARL2 in mitochondria. This result is consistent with ARL2 localizing to the matrix but it is likely that ARL2 is also present in the IMS. Increasing concentrations of digitonin selectively permeabilized outer and inner mitochondrial membranes and ARL2 staining was seen only after permeabilization of the inner mitochondrial membrane, the same condition required to visualize HSP60, a well-documented matrix protein. In contrast, cytochrome c (an IMS protein) staining of mitochondria was seen at lower concentrations of digitonin. The digitonin permeabilization approach does not rule out the presence of ARL2 in the IMS if that pool of the GTPase is not fixed well by paraformaldehyde. Thus, we conclude that a pool of mitochondria-associated ARL2 is in the matrix, though we cannot exclude the possibility that some ARL2 is also in the IMS. Indeed, we believe there *is* a pool of ARL2 in the IMS, based upon our earlier sub-mitochondria fractionation data [13] and more recent techniques that combine proteomics with spatially restricted protein tagging [15]; each of which concluded that ARL2 is in the IMS. Because ARL2 is present in mitochondria in amounts that preclude a stoichiometric binding to any of the complexes of the electron transport chain or transporters and is not found in any stable complexes, it is likely that ARL2 actions in the matrix are catalytic or regulatory and transient in nature, rather than as a stoichiometric component of a complex [62]. How then does the depletion of ARL2 cause these mitochondrial defects? We believe the best model is that ARL2 is involved in remodeling of the inner mitochondrial membrane, likely at crista junctions [63], [64]. Knockdown of either of the inner membrane proteins ChChd3 [49] or ChChd6 [50] results in mitochondrial fragmentation, perinuclear clustering, and loss of cell proliferation [49], [50] and ATP levels. ChChd3 siRNA also decreased cell proliferation without increasing apoptosis, again similar to ARL2 siRNA. The two ChChd proteins bind to each other and to Mitofilin, a core component of the MINOS [65] or MitOS [66], [67], [68], [69] complex that is key to crista junction formation and stabilization. The model for ARL2 affecting inner membrane remodeling also derives from analogy to the actions of the ARF (and ARL1) proteins, which are intimately involved in vesicle biogenesis and membrane remodeling at the Golgi and endosomes [70], [71], [72], [73], [74]. Thus, the intimate contact of ARF family members with membranes [75], ability to alter membrane curvature, their recruitment of other proteins with similar membrane sensing and modifying activities, and ability to alter localized phospholipid metabolism may be a more widespread feature of the ARF family than previously appreciated. These are very challenging issues to explore on the inner mitochondrial membrane, but research in this area is increasingly active and productive, since the identification of the MINOS complex. The hypothesis that ARL2 may be a component of the MINOS complex or regulate cristae morphology is currently under investigation in our lab.

When our latest observations are combined with earlier studies, we conclude that ARL2 is an important component of several cellular processes, including (i) regulation of ATP levels in mitochondria, likely in the matrix, (ii) regulation of mitochondrial fission and motility, (iii) at centrosomes, in concert with cofactor D, to regulate the growth of microtubules and mitotic spindles [10], [11], [22], [47], [76], (iv) in the nucleus to regulate STAT3 and perhaps other transcriptional responses [12], and in the cytosol to (v) regulate the folding of tubulin heterodimers [2], [3], and (vi) the shuttling and release of farnesylated proteins [24]. With the dissection of these different functions of ARL2 and the growing list of reagents allowing their clear resolution we are poised to understand the mechanisms of these actions at the molecular level, though some of them are expected to be challenging to document due to the limited understanding of the process itself (e.g., crista junction regulation). The fact that ARL2 is linked to so many different essential cellular functions and increasingly to human diseases drives further exploration into the mechanistic details of each of these actions.

## Supporting Information

Figure S1 **ARL2 and ARL2[T30N] are expressed when co-transfected with GFP-DRP1[K38A].** HeLa cells were co-transfected with either GFP or GFP-DRP1[K38A] and empty vector, ARL2, or ARL2[T30N]. Cells were harvested 24 hours later, lysed, and analyzed by immunoblot. The membrane was cut and probed for ARL2, GFP (for DRP1 expression), and actin (as a loading control).This experiment was done twice with similar results.
(TIF)

Figure S2 **Mitochondrial ARL2 staining is retained after cBid treatment.** HeLa cells were incubated in 0.2% saponin for 5 minutes, followed by a 20 minute incubation with 10 nM cBid or vehicle control. Cells were then fixed and immunostained for cytochrome c (top panels) or ARL2 (bottom panels). Cytochrome c staining is clearly lost after treatment with cBid (upper right panel), but ARL2 staining is retained (bottom lower panel).
(TIF)

Figure S3 **ELMOD2 mitochondrial staining is competed by purified, recombinant ELMOD2.** COS7 cells were fixed in 4% paraformaldehyde, permeabilized with 0.1% Triton X-100, and stained with pre-immune serum (left panels), immune serum (middle panels), and immune serum competed with recombinant ELMOD2 (right panels). Cells were also stained for cytochrome c as a mitochondrial marker.
(TIF)

Figure S4 **ELMOD2 localizes to the mitochondrial matrix.** HeLa cells were fixed in 4% paraformaldehyde prior to permeabilization in either 0.02% (two upper rows) or 0.1% (lowest row) (w/v) digitonin for 10 minutes at room temperature. Cells were then processed for imaging using dual labeling for ELMOD2 (green) and either cytochrome c (top row, middle panel) or HSP60 (lower two rows, middle panels), as markers of the IMS and matrix, respectively.
(TIF)
