# Supplementary figures and images for: The ARL2 GTPase Is Required for Mitochondrial Morphology, Motility, and Maintenance of ATP Levels
Source: PLoS One. 2014 Jun 9;9(6):e99270. doi: 10.1371/journal.pone.0099270 (PMC4050054; doi:10.1371/journal.pone.0099270)

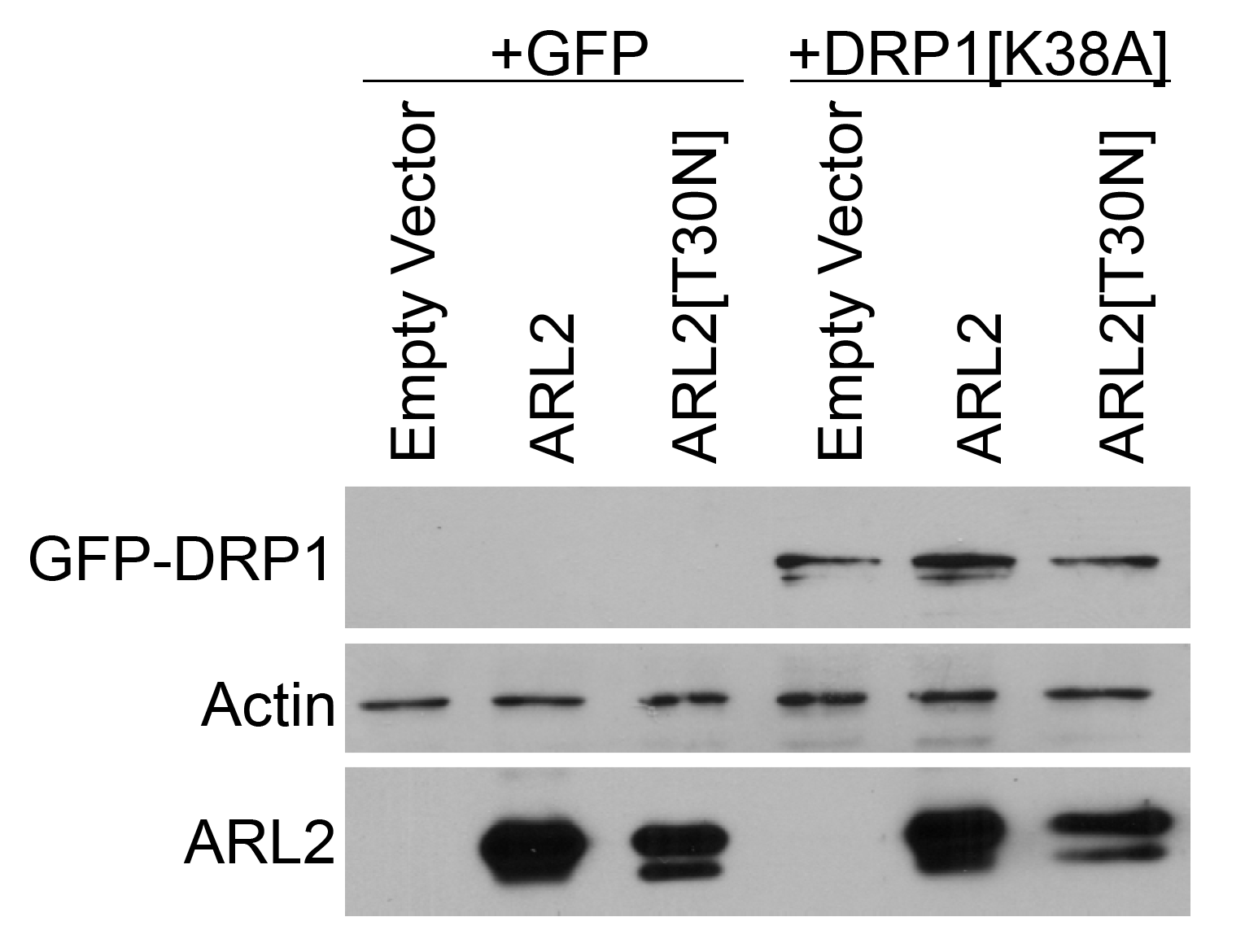

Supplement: Figure S1 — ARL2 and ARL2[T30N] are expressed when co-transfected with GFP-DRP1[K38A]. HeLa cells were co-transfected with either GFP or GFP-DRP1[K38A] and empty vector, ARL2, or ARL2[T30N]. Cells were harvested 24 hours later, lysed, and analyzed by immunoblot. The membrane was cut and probed for ARL2, GFP (for DRP1 expression), and actin (as a loading control).This experiment was done twice with similar results. (TIF) [file pone.0099270.s001.tif]

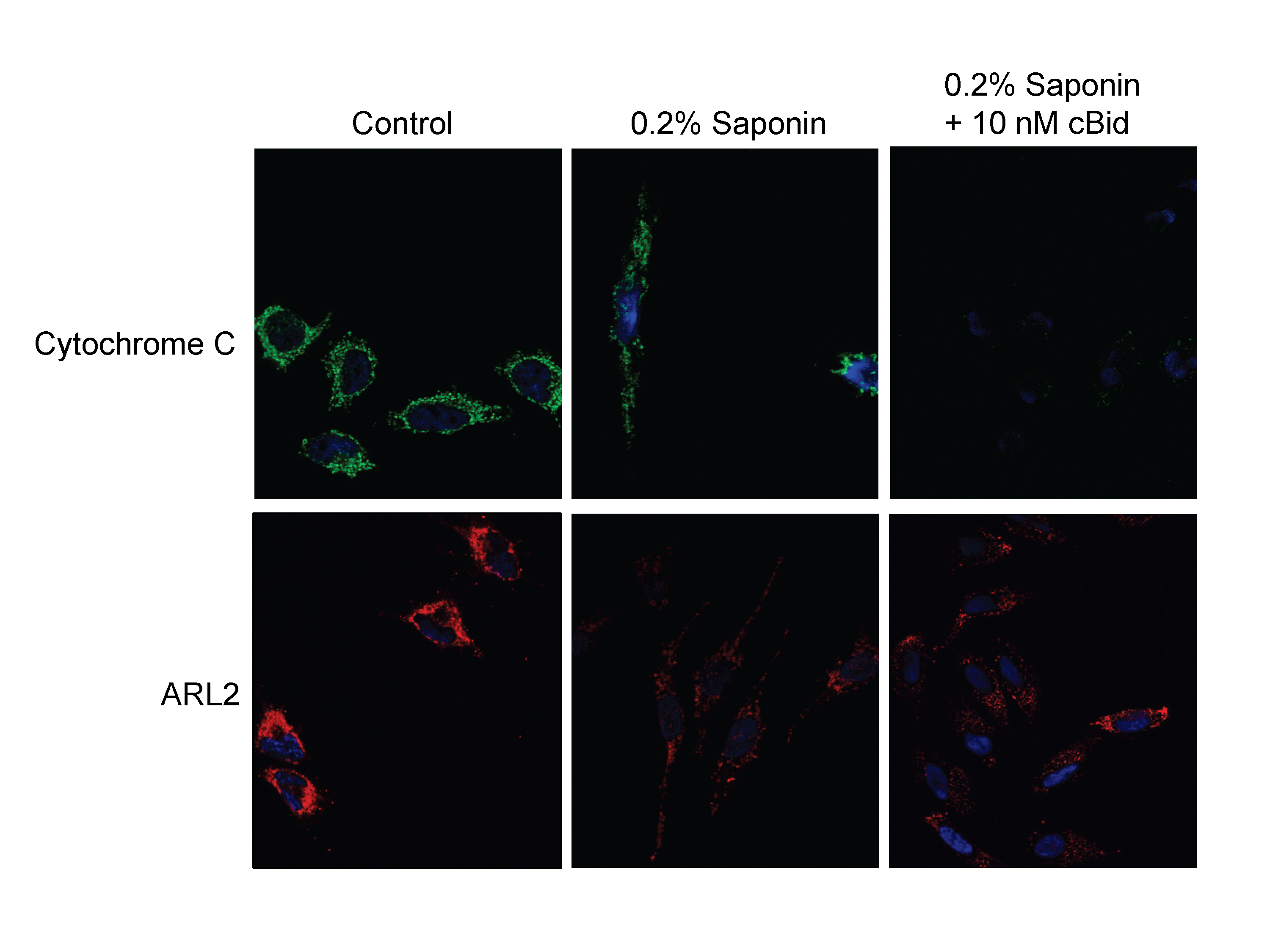

Supplement: Figure S2 — Mitochondrial ARL2 staining is retained after cBid treatment. HeLa cells were incubated in 0.2% saponin for 5 minutes, followed by a 20 minute incubation with 10 nM cBid or vehicle control. Cells were then fixed and immunostained for cytochrome c (top panels) or ARL2 (bottom panels). Cytochrome c staining is clearly lost after treatment with cBid (upper right panel), but ARL2 staining is retained (bottom lower panel). (TIF) [file pone.0099270.s002.tif]

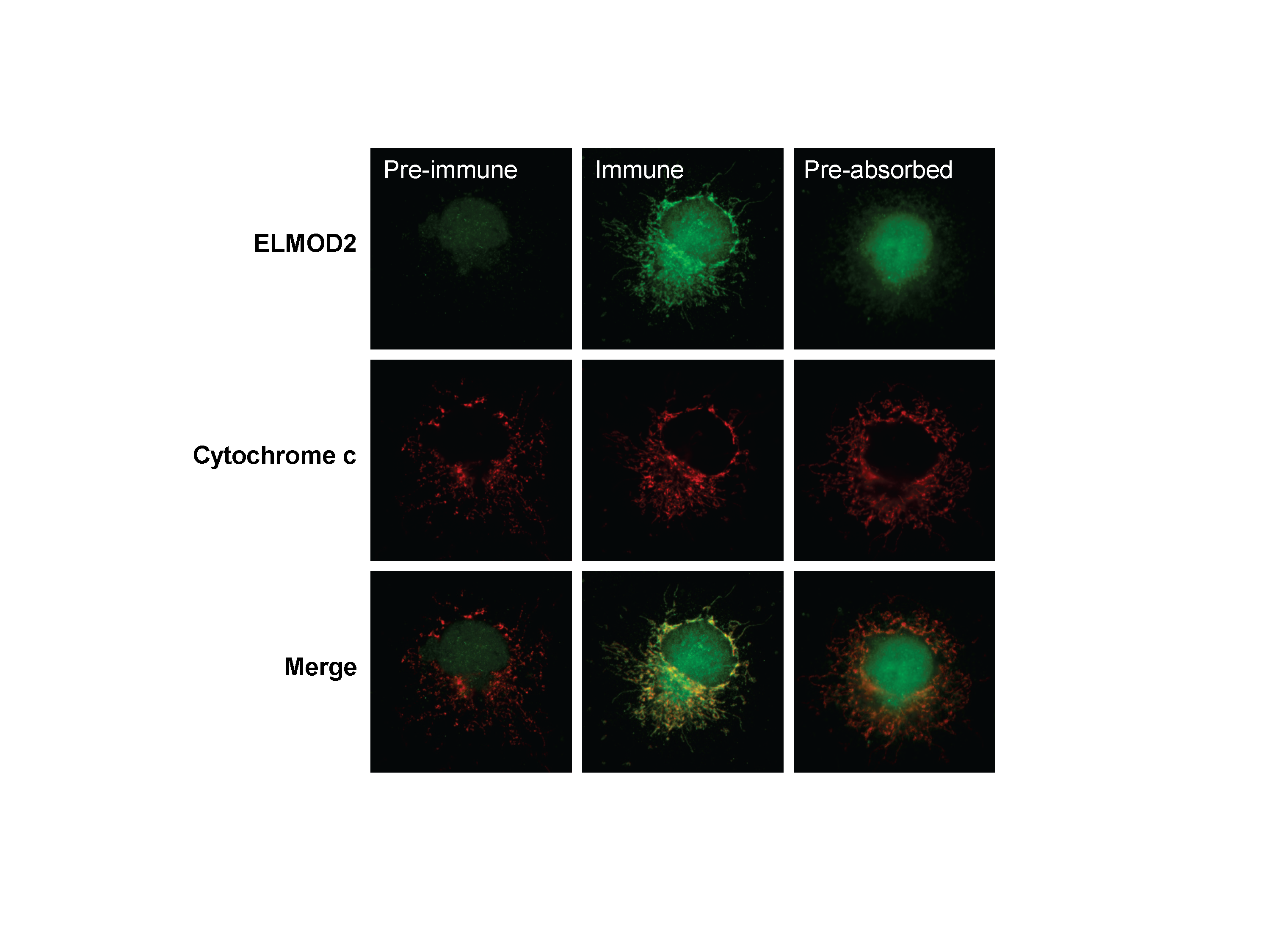

Supplement: Figure S3 — ELMOD2 mitochondrial staining is competed by purified, recombinant ELMOD2. COS7 cells were fixed in 4% paraformaldehyde, permeabilized with 0.1% Triton X-100, and stained with pre-immune serum (left panels), immune serum (middle panels), and immune serum competed with recombinant ELMOD2 (right panels). Cells were also stained for cytochrome c as a mitochondrial marker. (TIF) [file pone.0099270.s003.tif]

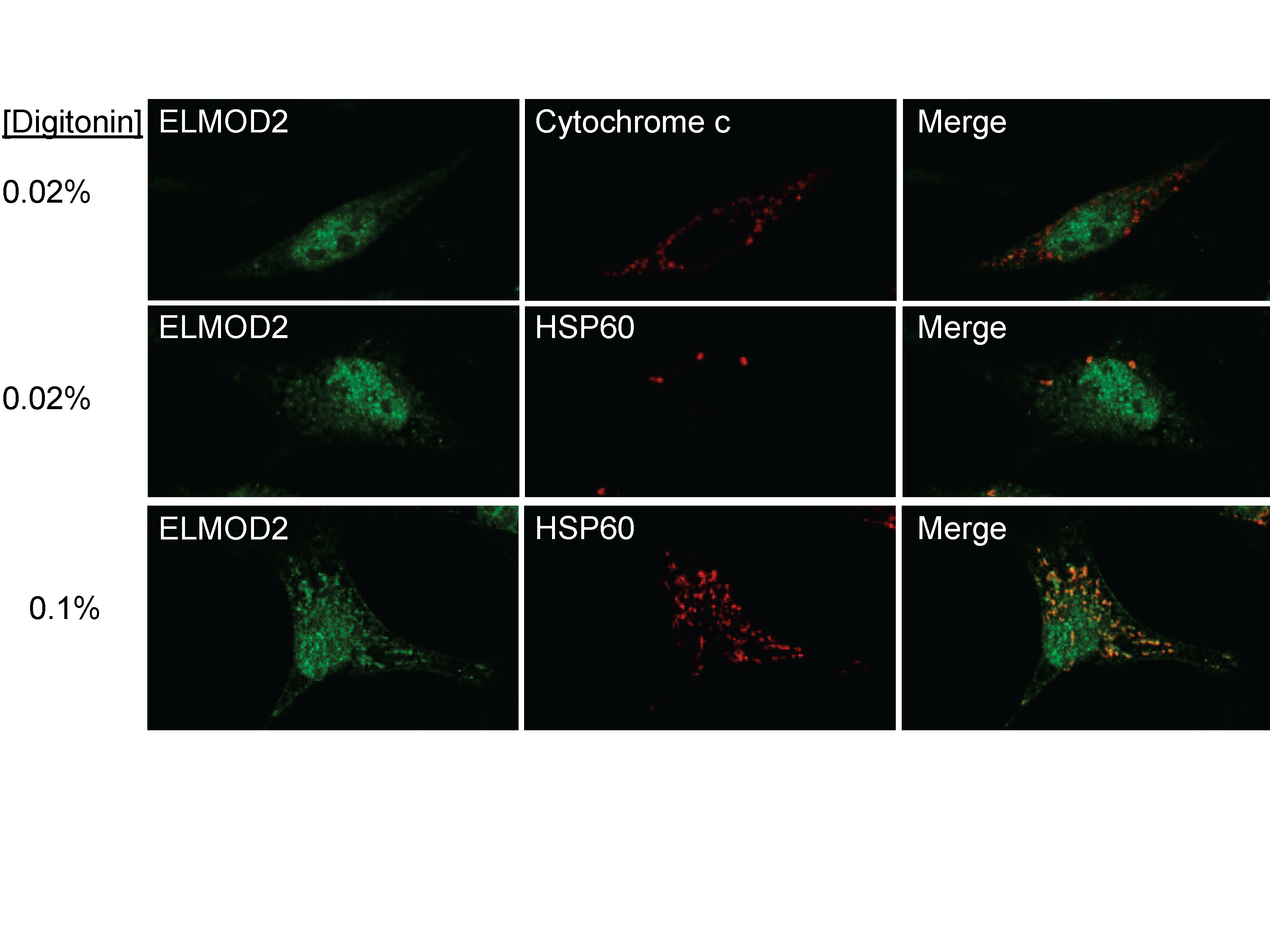

Supplement: Figure S4 — ELMOD2 localizes to the mitochondrial matrix. HeLa cells were fixed in 4% paraformaldehyde prior to permeabilization in either 0.02% (two upper rows) or 0.1% (lowest row) (w/v) digitonin for 10 minutes at room temperature. Cells were then processed for imaging using dual labeling for ELMOD2 (green) and either cytochrome c (top row, middle panel) or HSP60 (lower two rows, middle panels), as markers of the IMS and matrix, respectively. (TIF) [file pone.0099270.s004.tif]
